# The Effect and Possible Mechanism of Intradiscal Injection of Simvastatin in the Treatment of Discogenic Pain in Rats

**DOI:** 10.3389/fnins.2021.642436

**Published:** 2021-03-17

**Authors:** Xiaodong Huang, Changkun Zheng, Weiheng Wang, Xiaojian Ye, Chia-Ying Lin, Zenghui Wu

**Affiliations:** ^1^Department of Orthopedics, The Third Affiliated Hospital of Guangzhou Medical University, Guangdong, China; ^2^Department of Orthopaedic Surgery, College of Medicine, University of Cincinnati, Cincinnati, OH, United States; ^3^Department of Orthopaedics, Shanghai Changzheng Hospital, Shanghai, China; ^4^Department of Orthopaedics, Fuzhou Second Hospital Affiliated to Xiamen University, Fujian, China

**Keywords:** simvastatin, discogenic low back pain, cold sensitivity, NF-κB – Nuclear factor kappa B, GAP43=growth-associated peptide 43

## Abstract

To study the effect of intradiscal injection of simvastatin on discogenic pain in rats and its possible mechanism, 30 adult female rats were used in this experiment. Twenty rats were randomly divided into sham operation group (Control group), intervertebral disk degeneration group (DDD group), intervertebral disk degeneration + hydrogel group (DDD + GEL group), and intervertebral disk degeneration + simvastatin group (DDD + SIM group). The mechanical pain threshold and cold sensation in rats were measured. The contents of NF-kappa B1, RelA, GAP43, SP, CGRP, TRPM 8, IL-1β, and TNF-α in the intervertebral disk (IVD), the corresponding contents of dorsal root ganglion (DRG) and plantar skin GAP43 and TRPM 8 were quantitatively detected by PCR. The corresponding IVDs were stained to detect their degeneration. There was no significant difference in the mechanical pain threshold between the groups at each time point. From the first day to the 8th week after surgery, the cold-sensing response of the DDD group was significantly higher than that of the Control group (*P* < 0.05). At 7 and 8 weeks postoperatively, the cold-sensing response of the DDD + SIM group was significantly lower than that of the DDD + GEL group (*P* < 0.05). The levels of NF-κB1, RelA, GAP43, SP, CGRP, TRPM8, IL-1β, and TNF-α in the IVD of DDD + SIM group were significantly lower than those in DDD group (*P* < 0.05). The content of GAP43 and TRPM8 in rat plantar skin decreased significantly and TRPM8 in DRG decreased significantly (*P* < 0.05).

## Introduction

Lower back pain (LBP) is a common musculoskeletal disease and one of the main causes of disability worldwide (Murray et al., 2013). LBP is pain confined to the area below the ribs and the lower buttocks folds, with or without leg pain (van Tulder et al., 2002; van Tulder and Koes, 2006). The etiology of LBP is characterized by diversity and complexity. At least 40% of LBP cases may be related to intervertebral disk (IVD) degeneration, that is, discogenic low back pain (DLBP; Barrick et al., 2000).

At present, the most commonly used treatment methods for DLBP include conservative treatment (physical therapy and oral anti-inflammatory drugs, etc.), minimally invasive interventional techniques (epidural injections and radiofrequency ablation techniques, etc.) to conventional surgical treatment (Di Martino et al., 2013). Each of these treatment methods aims to alleviate related clinical symptoms, none of them target the specific underlying pathophysiology itself or reverse its degeneration process. With the emergence of recombinant therapeutic proteins (Masuda, 2008), people began to advocate bioremediation or regeneration of degenerative IVD. This new type of treatment seems promising because it helps synthesize the matrix molecules that make up the IVD structure, and may also help prevent matrix degradation and/or cell death, thereby preventing the progression of IVD degeneration.

At present, many studies have shown that many growth factors, such as transforming growth factor-β (TGF-β), growth differentiation factor 5 (GDF-5), fibroblast growth factor (FGF), and insulin-like growth factor-1 (IGF-1) have biological repair potential, especially have been proved to be very effective in promoting the anabolism of IVD cells (Gruber et al., 1997; Chujo et al., 2006). It has been confirmed that injection of growth factor GDF-5 or OP-1 into degenerative IVDs can repair abnormal IVD matrix *in vivo* (Chujo et al., 2006; Imai et al., 2007). However, there are still some problems with these recombinant human growth factors, such as the vascular growth caused by the injection of growth factor into IVD and the need to give super physiological dose to obtain the effectiveness of the drug. In addition, the price of these recombinant proteins is very expensive, which makes patients and doctors fear.

The ideal treatment will not produce harmful effects or damage to tissues, but will repair and/or regenerate damaged tissues. At the same time, it has the advantages of cost-effectiveness, minimally invasive, sustainable, safe and easy to obtain. Studies have shown that bone morphogenetic proteins (BMP-2, -5, -6, -8, -9, and -14) have the above advantages (Reddi, 1994; Snelling et al., 2010). Mundy et al. (1999) tested more than 30,000 compounds to determine their effects on BMP-2 gene expression. They found that statins, an HMG CoA reductase inhibitor, specifically increased BMP-2 mRNA expression in bone cells *in vitro*. At present, simvastatin treatment of DLBP may be through up regulating the expression of bmp-2a, promoting the expression of chondrocyte phenotype, thus delaying the degeneration of IVD (Zhang and Lin, 2008).

Discogenic low back pain is closely related to IVD degeneration (Adams, 2004). IVD degeneration is an inevitable physiological and pathological process with age and mechanical stimulation. Studies have shown that IVD degeneration begins after the age of 14. Anatomic studies have shown that there are nerve fibers growing into degenerative IVDs, but not all of them can induce DLBP. DLBP is closely related to two factors: nerve fiber growth and IVD environment. It has been shown that the growth of nerve fibers is the histological basis of DLBP. It is generally believed that the normal IVD is innervated by the sinus vertebral nerve and sympathetic nerve, and the nerve fibers only exist on the surface of the outer annulus fibrosus. During IVD degeneration, IVD cells exposed to proinflammatory cytokines (such as IL-1 β, etc.) can increase the secretion of many different signal molecules (neurotrophic factors) (Abe et al., 2007; Gruber et al., 2012). These neurotrophic factors include nerve growth factor (NGF), neurotrophic factor 3 (NT3), nerve fiber protein 1 and 2 and S100B (Kalcheim et al., 1992; Purmessur et al., 2008). In addition, the decrease of glycosaminoglycan content in the IVD may lead to the growth of capillaries and nerve fibers from the outer annulus fibrosus to the inner annulus fibrosus or even into the nucleus pulposus (Buirski and Silberstein, 1993; Bogduk et al., 2013). The growth of nerve fibers is related to the distribution of peripheral capillaries, which can nourish and protect nerve fibers. Binch et al. (2015) have also shown that there are nerves and blood vessels growing into the degenerative IVD, and nerve fibers are more abundant than blood vessels. The degeneration of the IVD environment changes, increased inflammatory factors and oxidative stress response, stimulate new nerve, is an important cause of DLBP (Garcia-Cosamalon et al., 2010). Many studies have shown that the reduction of disk inflammation and/or the ablation of injurious nerves can improve the clinical symptoms of patients with discogenic low back pain (Peng et al., 2005). However, the mechanism of DLBP needs further study.

At present, studies have shown that simvastatin can delay the degeneration of IVD, but its effect on DLBP and its specific mechanism have not been reported (Zhang and Lin, 2008). This study is to study the therapeutic effect of simvastatin on DLBP in rats, and to explore the effect and mechanism of simvastatin on intervertebral nerve ingrowth. The specific therapeutic mechanism of simvastatin was discussed from two aspects: the influence of simvastatin on the nerve ingrowth in IVD and the influence on the environment of IVD.

## Materials and Methods

### Experimental Animals

Thirty 3-month-old female Sprague Dawley rats (180–230 g) were purchased from the animal house of University of Cincinnati. Each cage is housed with two rats. These rats are housed in a specific room. The air filtration rate is 10–20 times per hour; the temperature is 20–26°C, the humidity is 40–70%, daily illuminate for 12 h (08:00–20:00) and then 12 h dark fluorescent light. These rats were placed in an environment where they could eat and drink freely, and they had to adapt to the environment for at least 2 weeks before surgery. All animal operations in this experiment were approved by the Animal Ethics Committee of University of Cincinnati.

### Establishment of Rat DLBP Model

**FIGURE 1:**
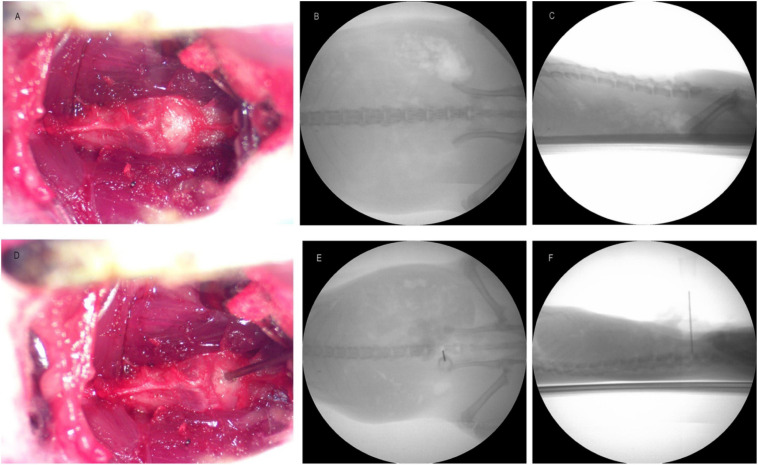
Panel **(A)** is the image of intervertebral disk before puncture. Panels **(B,C)** are the X-ray images of anterior and lateral position before puncture. Panel **(D)** is the picture of intervertebral disk during puncture. Panels **(E,F)** are the pictures of X-ray at the time of puncture.

Twenty-five rats were used to establish DLBP model. Five percent isoflurane was used to induce anesthesia and 2% isoflurane was used to maintain anesthesia. The back of rats was placed on the heating pad, and then the abdominal skin was prepared and disinfected. A 2–3 cm longitudinal incision was made with the intersection of xiphoid process, pubic symphysis, and bilateral iliac crest as the center. The abdominal muscle tissue under the skin was cut to expose the abdominal cavity. The intestines were gently moved to the upper, lower, left, and right sides of the abdominal cavity with sterile cotton balls soaked in sterile saline solution. On the left side of the abdominal aorta, the midline connective tissue separating the left and right psoas muscles was carefully identified, and the vertebral body or IVD tissue below it was exposed. Touching or disturbing spinal nerve roots or damaging blood vessels or muscles was carefully avoided. The L4/5 IVD (Figures 1A–C) was exposed, and then the IVD was vertically punctured with 23G needle at the center of IVD (Figure 1D). The puncture needle was confirmed to be located in the space center of IVD by C-arm machine anteroposterior and lateral inspection (Figures 1E,F), and the needle was kept in the IVD for 1 min. The needle was rotated left and right three times prior to its slow removal. Take out the puncture needle slowly, and tissue obtained was examined microscopically to verify its origin as nucleus pulposus. Cotton balls were taken out and the internal organs of abdominal cavity were put back in place. The abdominal muscles were sutured with 6-0 coated Vicryl (polygactin 910) suture (J489G; Ethicon, Cincinnati, OH, United States). The skin incision was sutured with silk 6-0 suture (706g; Ethicon). Keep animals warm and monitor until awake from anesthesia. The animals were continuously monitored for 3 days and then twice a week.

### Animal Grouping and Treatment of DLBP

Two weeks after the establishment of the rat DLBP model. Based on body weight, 20 rats were divided into four groups by randomization in the BioBook system (IDBS), Control group, DDD group, DDD + GEL group, DDD + SIM group. Five percent isoflurane was used to induce anesthesia and 2% isoflurane was used to maintain anesthesia. The L4/5 IVD was exposed along the original incision. In the control group, only L4/5 IVD was exposed; in the DDD group, the ventral side of the L4/5 IVD was slowly punctured with a 25G needle; in the DDD + GEL group, the L4/5 IVD was punctured and the hydrogel was injected through a micro syringe; In the DDD + SIM group, simvastatin (3 mg/mL) was injected through a microsyringe while puncturing the L4/5 IVD. With hemostatic forceps, the depth of the puncture needle into the IVD was 5 mm, the volume of injection into each IVD was 10 μL, and the injection time was 1 min. After the injection, the needle was kept in the IVD for 1 min, and then withdrawn slowly to prevent fluid leakage. The incision was closed as before. Animals were kept warm and monitored until fully recovered from anesthesia. The animals were continuously monitored for 3 days and then twice a week.

### Detection of Pain and Temperature in Both Lower Limbs of Rats

Previous articles described the testing process in detail (Song et al., 1999). Animals were tested every other day before surgery over a period of 5 days (three tests; the average value was used as the baseline on the 0th day). Behavioral tests were performed on the 1st day, 1, 2, 3, 4, 5, 6, 7, and 8 weeks after the operation. In order to avoid prejudice, the person conducting the behavioral test does not know the grouping of the experiment. In the test, the rat was placed in a plastic transparent glass box and allowed to acclimate for 30 min, and then the test was performed. Mechanical allodynia and hyperalgesia were monitored by applying von Frey filament to the planter surface of the hind paw very slowly, and then pressing at a 90° angle until it bends (Chaplan et al., 1994). The filament was applied to the hind paw for 5 s, and then slowly removed. If a paw withdrawal response was observed the animal was tested again with a filament reduced by one level. If no response was observed the strength of the filament was increased by one level. The process was repeated until the first transition from paw withdrawal to no paw withdrawal (or vice versa) was observed. Testing was carried out six times every 5 min per animal.

Tactile detection was used as an additional measurement of allodynia, this involved the use of a strand of cotton on a cotton swab to gently sweep from the front to the back of the plantar surface of the hind paw. To assess the presence or absence of a rapid withdrawal response to usually harmless mechanical stimuli (mild tactile allodynia). Normal animals do not respond to this stimulus. Data were quantified by noting percentage of animal responding.

The cold sensitivity score was the response to a drop of acetone on the ventral surface of the hind paw (Xu and Brennan, 2010). When observed, the positive reaction included several quick flicks of the paw and/or licking and shaking of the paw; walking motion was not a positive reaction. Data were quantified by noting percentage of animal responding.

### Voluntarily Accessed Static Incapacitance Chamber Device

The analog weight information obtained from the load cell that is updated every 100 ms is converted into digital data by the on-board analog-digital converter. The local microprocessor averages the weight data in a user-defined time interval, and the average weight data is transmitted to the host computer through the Windows analog serial port on the USB port. The correct position of the animal was determined by the infrared beam circuit breaker detector located under the water source. The detector can only work normally when the animal positions itself on the weighing platform. Once captured by the host computer, the weight data and time stamps on the right and left were organized into text files and saved. The analysis software developed in Excel (Microsoft, Seattle, WA, United States) reads the text data, applies the data filter selected by the user, and writes the processed data to the output column of the spreadsheet. The numbers were generated from the output text file in Excel. The current voluntarily accessed static incapacitance chamber (VASIC) model allows the detection sensitivity of each foot pad to be 0.2–800 g. The internal chamber and software calibration are designed and optimized to suit the size of the rat, which can be used for rats with a weight range of 70–500 g.

### Histological Examination of Intervertebral Disk

Two weeks after the establishment of the rat DLBP model, five normal rats and five DLBP rats were anesthetized with 2% pentobarbital through the abdominal cavity and then perfused. Firstly, the animals were perfused with 0.1 μM phosphate buffer solution until the liquid flowing out was transparent, and then perfused with 4% PFA for 20 min. Fluid enters the left ventricle and exits the right atrium. The L4/5 disks of the Control group and DDD group were separated by observation with an upright microscope. Tissues were fixed in in formaldehyde for at least 48 h prior to paraffin embedding and sagittal sectioning (4 μm thick). Hematoxylin and eosin (H&E) staining, Alcin Blue staining, and Safranin O staining were performed through the Pathology Research Center and an Olympus optical microscope used for qualitative analysis. At 8 weeks after operation, the same method was used to perform H&E staining and analysis on the IVDs in the Control group, DDD group, DDD + GEL group, and DDD + SIM group.

### Quantitative qRT-PCR of Intervertebral Disk, DRG, and Plantar Skin

At 8 weeks after operation, five rats in each group were killed. The intact L4/5 IVD, bilateral DRG and bilateral dorsal plantar skin were observed under an upright microscope. The relative mRNA levels of pain related genes were analyzed by real-time quantitative PCR (QRT PCR). Table 1 is the primer sequence of the target gene. Total RNA was extracted by Trizol reagent. The cDNA from the samples was processed by superscript III cellsdirect cDNA synthesis system (Invitrogen, Grand Island, NY, United States). Mpx3005 instrument (stratagene, Agilent Technologies, Inc., Santa Clara, CA, United States) and faststart universal SYBR Green Master Mix (Roche Applied Science, Indianapolis, IN, United States) were used to detect the expression of GAP43, SP, CGRP, TRPM8, IL-1 β, and TNF-α in IVD, and the expression of gap43 and TRPM8 in skin and DRG were detected by quantitative PCR. The primers for rat specific genes are as follows. After 50 amplification cycles, the analysis of the chain breaking curve was carried out. Samples were analyzed independently using linreg PCR software, which directly determined the baseline, CQ value and amplification efficiency from the amplification curve.

**TABLE 1:** Primer sequences of target genes detected by PCR.

Name	Primer	Sequence (5′–3′)
GAP43	Forward	TGTGCTGTAT GAGAAGAACC
	Reverse	GATAAGGCTC ATAAGGCTG
NF-kB1	Forward	TGA ATC CCC CTG AGA AAG AA
	Reverse	GTT ATC CTG AAA CCC CAC ATC
RelA	Forward	GCT GTT TGG TTT GAG ACA TC
	Reverse	TCT GCC CTC CTG ACT CTA CT
SP	Forward	CTTCAGCAAA GCACAGTGTT
	Reverse	GACAGAAAGG CTGCTGTGAGG
TRPM8	Forward	GCACTCCTT ACCTTTGTCTG
	Reverse	GACTTGGATGT GGAACTCC
CGRP	Forward	TTCTTGGTACTGAAACCCTTC
	Reverse	GAA TCA CAT ACA ACA CGA TGC
TNF-α	Forward	CACGATGCACCTGTAGATCA
	Reverse	GTTGCTCCATATCCTGTCCCT
IL-1β	Forward	GATGGACTCACCAGGTGAG
	Reverse	CTCATGGTGTCCTTTCCAGG

### Statistical Analysis

PASW statistics 21.0 (SPSS Inc.) software was used for data analysis and graph pad prism 8 software was used for mapping. All experimental data were expressed as mean ± SD. One way ANOVA was used for intra group comparison, while variance of repeated measurement design was used for inter group comparison. The difference was statistically significant when *P* < 0.05.

## Results

### Validation of DLBP Model

**FIGURE 2:**
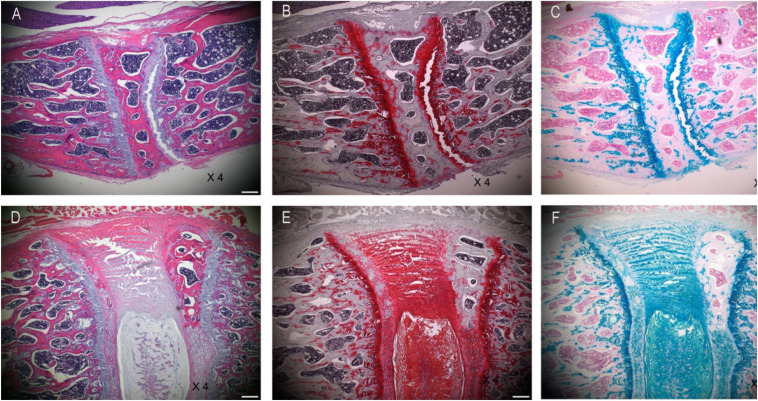
Histological changes validate of DLBP model. Panels **(A,D)** are H&E staining of intervertebral disk. Panels **(B,E)** are Safranin O staining of intervertebral disk. Panels **(C,F)** are Alcin Blue staining of intervertebral disk.

Two weeks after the establishment of DLBP, the histological staining of normal rats and DLBP rats is as follows. H&E staining is used to observe the morphology of tissues and the number of cells. H&E staining showed that the annulus fibrosus tissue of the IVD was disorderly arranged after acupuncture, and the number of nucleus pulposus cells and annulus fibroblasts decreased (Figures 2A,D). Alcin Blue staining and Safranin O staining are mainly used to observe the bone formation of tissues. Alcin Blue staining shows that the annulus fibrosus and nucleus pulposus of the IVD are stained blue after acupuncture (Figures 2C,F). Safranin O staining showed that the annulus fibrosus and nucleus pulposus of the IVD were stained red after acupuncture (Figures 2B,E). These results indicate that punctate lesion can cause disk degeneration after 2 weeks.

### Effects of Intradiscal Simvastatin on Mechanical Pain and Cold Sensation in Animals

**FIGURE 3:**
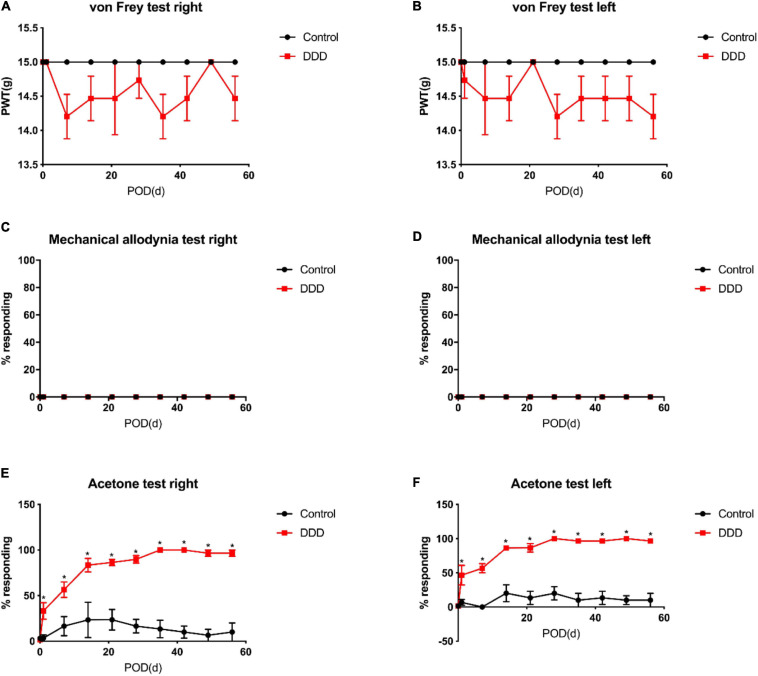
The mechanical pain and cold response of animals. Panel **(A)** is the result of von-Frey of the right lower limb of the rat. Panel **(B)** is the result of the von-Frey of the left lower limb of the rat. Panel **(C)** is the result of the tactile response of the right lower limb of the rat. Panel **(D)** is the result of the tactile response of the left lower limb of the rat. Panel **(E)** is the result of the cold response of the right lower limb. Panel **(F)** is the result of the cold response of the left lower limb of the rat; all data are expressed as mean ± standard deviation, *P < 0.05 vs. DDD group (*n* = 5). PTW, paw withdrawal threshold; POD, post-operation days.

**FIGURE 4:**
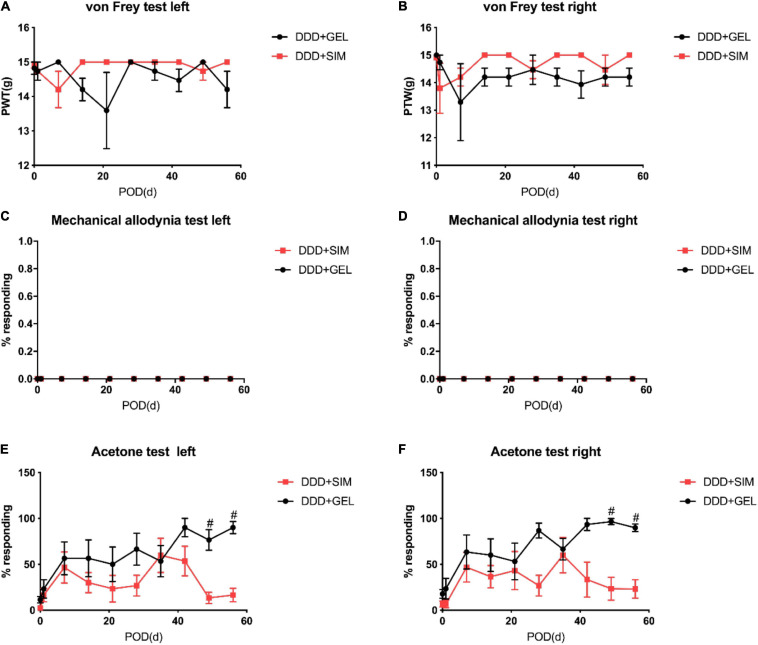
The effect of intradiscal injection of simvastatin gel on mechanical pain and cold response in animals. Panel **(A)** is the result of von-Frey of the left lower limb of the rat. Panel **(B)** is the result of the von-Frey of the right lower limb of the rat. Panel **(C)** is the result of the tactile response of the left lower limb of the rat. Panel **(D)** is the result of the tactile response of the right lower limb of the rat. Panel **(E)** is the result of the cold response of the left lower limb. Panel **(F)** is the result of the cold response of the right lower limb of the rat; all data are expressed as mean ± standard deviation, ^#^*P* < 0.05 vs. DDD + SIM group (*n* = 5).

Studies have shown that we can measure the change of pain threshold by observing the response of hind paw to mechanical, thermal or cold stimulation (Xie et al., 2016). The results of von Frey experiment showed that the pain threshold of both lower limbs in the control group remained at the same level as that before operation, which indicated that the operation would not affect the lower limb pain of animals; the plantar pain threshold of both lower limbs in DDD group and DDD + GEL group decreased sharply from the first day after operation, and then kept at a low level at 8 weeks after operation. The plantar pain threshold of DDD + SIM group decreased from the first day after operation, then increased slowly, and tended to be stable after 3 weeks (Figures 3A,B). The pain threshold of DDD group was lower than that of control group at all time points after operation, but there was no significant difference between the two groups (*P* > 0.05, Figures 4A,B). The results of tactile test showed that there was no tactile positive reaction in DDD group, control group, DDD + GEL group and treatment group before and after operation, which indicated that intervertebral disc puncture would not cause changes in tactile response of animals (Figures 3C,D, 4C,D). The results of acetone test showed that the cold response of DDD group, DDD + GEL group and DDD + SIM group increased rapidly from the first day after operation, but the cold sense response of DDD + SIM group began to decrease at the fifth week (Figures 3E,F, 4E,F). From the first day after operation, the cold sense reaction of DDD group was higher than that of control group (*P* < 0.05, Figures 3E,F). At 7 and 8 weeks after operation, the cold response of DDD + SIM group was lower than that of DDD + GEL group (*P* < 0.05, Figures 4E,F). These results suggest that intradiscal injection of simvastatin can alleviate cold hypersensitivity.

### VASIC Device to Measure the Weight-Bearing Condition of Both Lower Limbs of Rats

**FIGURE 5:**
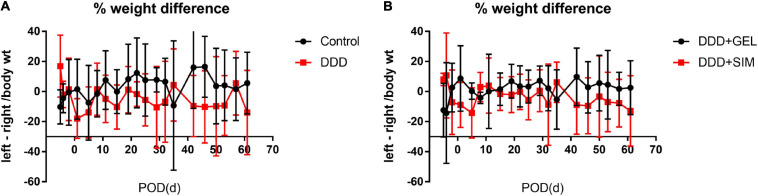
The effect of intradiscal injection of simvastatin on the weight bearing of the lower limbs of animals. Panels **(A,B)** is the result of the weight difference of the left and right lower limbs of the rat. All data are expressed as mean ± standard deviation (*n* = 5).

At all time points observed in the experiment, the weight of the left and right lower limbs of the rats in each group was basically the same, and the difference was not statistically significant (*P* > 0.05, Figures 5A,B). It shows that the DLBP animal model established by intervertebral disc puncture will not have symptoms of both lower limbs.

### The Effect of Intradiscal Injection of Simvastatin on Animal Intervertebral Disk Histology

**FIGURE 6:**
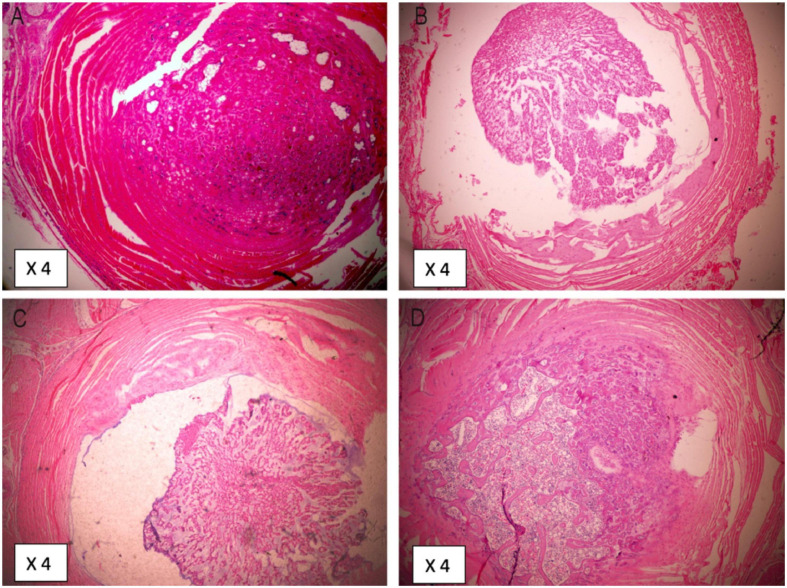
Histological changes 8 weeks after intradiscal injection of simvastatin. Panel **(A)** is the tissue from Control group. Panel **(B)** is the tissue from DDD group. Panel **(C)** is the tissue from DDD + GEL group. Panel **(D)** is the tissue from DDD + SIM group.

8 weeks after intradiscal intervention, the H&E staining of DLBP rats in each group is as follows. H&E staining results show that simvastatin can improve the integrity of the intervertebral disc (Figures 6A–D).

### The Effect of Intradiscal Injection of Simvastatin on the Content of Nerve Mediators in Animal Intervertebral Disks

**FIGURE 7:**
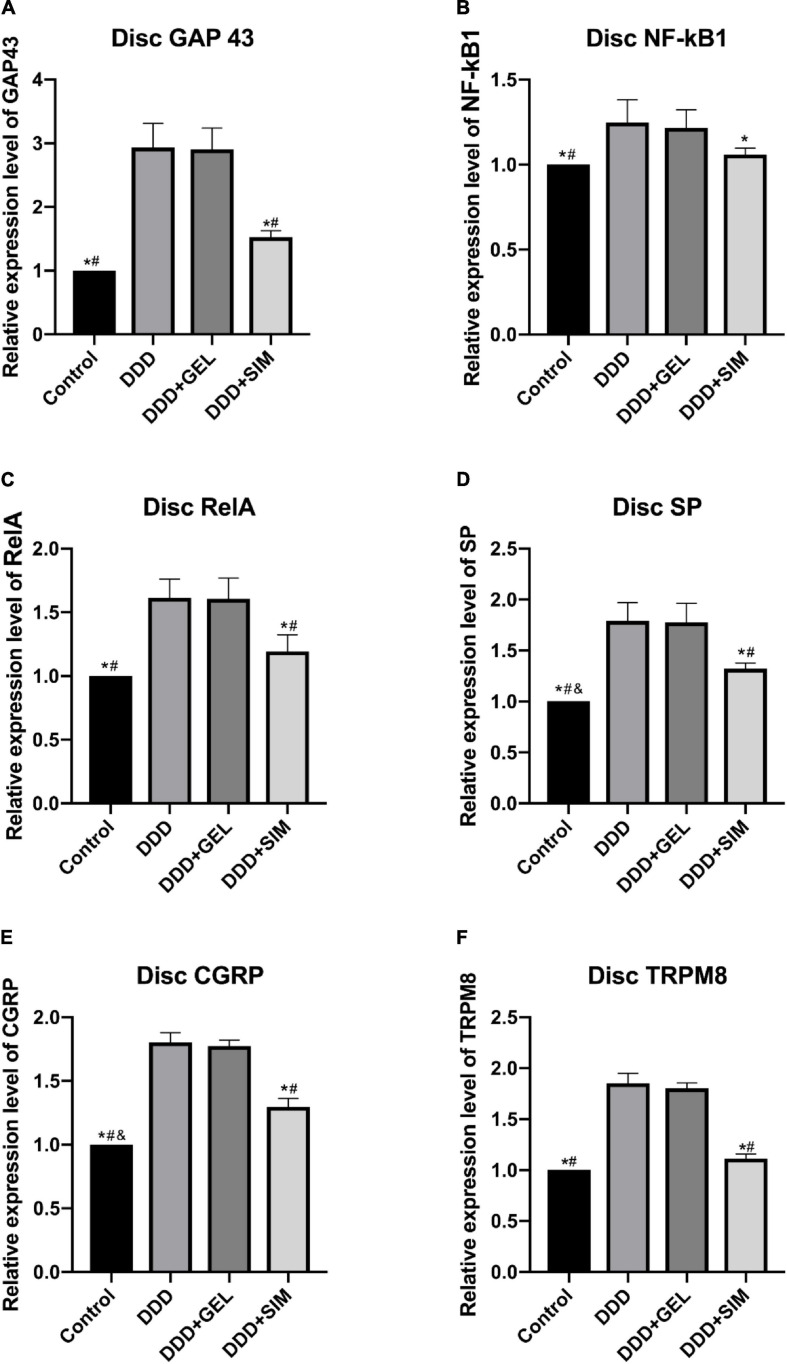
The effect of intradiscal injection of simvastatin on the content of nerve mediators in animal intervertebral disks. Panel **(A)** is the change of GAP43 of each group of intervertebral disk. Panel **(B)** is the change of NF-kB1 of each group of intervertebral disk. Panel **(C)** is the change of RelA of each group of intervertebral disk. Panel **(D)** is the change of SP of each group of intervertebral disk. Panel **(E)** is the change of CGRP of each group of intervertebral disk Situation. Panel **(F)** is the change of TRPM8 in each group of intervertebral disks; all data are expressed as mean ± standard deviation, *P < 0.05 vs. DDD group, ^#^*P* < 0.05 vs. DDD + GEL group, ^&^*P* < 0.05 vs. DDD + SIM group (*n* = 5).

The GAP43 (Growth Associated Protein-43) content of IVDs in the Control group (*P* < 0.0001, *P* < 0.0001) and DDD + SIM group (*P* = 0.0005, *P* = 0.0005) were lower than those in the DDD group and DDD + GEL group (Figure 7A). The content of NF-kB1(Nuclear Factor-Kappa B 1) in the IVD in the Control group was lower than that in the DDD group and the DDD + GEL group (*P* = 0.0127, *P* = 0.0236); the content of NF-kB1 in the IVD in the DDD + SIM group was lower than that in the DDD group (*P* = 0.0430) (Figure 7B). The RelA content of IVDs in the Control group (*P* = 0.0003, *P* = 0.0003) and the DDD + SIM group (*P* = 0.0020, *P* = 0.0023) were lower than those in the DDD group and DDD + GEL group (Figure 7C). The SP (Substance P) content of the IVD in the Control group was lower than that of the DDD group, DDD + GEL group, and DDD + SIM group (*P* = 0.0002, *P* = 0.0002, *P* = 0.0226); the SP content of the IVD in the DDD + SIM group was lower than that of the DDD group and DDD + GEL Both groups decreased (*P* = 0.0037, *P* = 0.0043, Figure 7D). The CGRP (Calcitonin Gene-Related Peptide) content of the IVD in the Control group was lower than that of the DDD group, DDD + GEL group and the DDD + SIM group (*P* < 0.0001, *P* < 0.0001, *P* = 0.0029); the CGRP content of the IVD in the DDD + SIM group was lower than that of the DDD group and DDD + GEL Both groups are reduced (*P* = 0.0002, *P* = 0.0002, Figure 7E). The levels of TRPM8 (Transient Receptor Potential Melastatin 8) in IVDs in the Control group (*P* < 0.0001, *P* < 0.0001) and DDD + SIM group (*P* < 0.0001, *P* < 0.0001) were lower than those in the DDD group and DDD + GEL group (Figure 7F). These data indicate that intradiscal injection of simvastatin can significantly reduce the nerve growth in the IVD (the content of GAP43 is significantly reduced), and it can also reduce the cold response (the content of TRPM8 is reduced).

### The Effect of Intradiscal Injection of Simvastatin on Animal DRG Nerve Mediators

**FIGURE 8:**
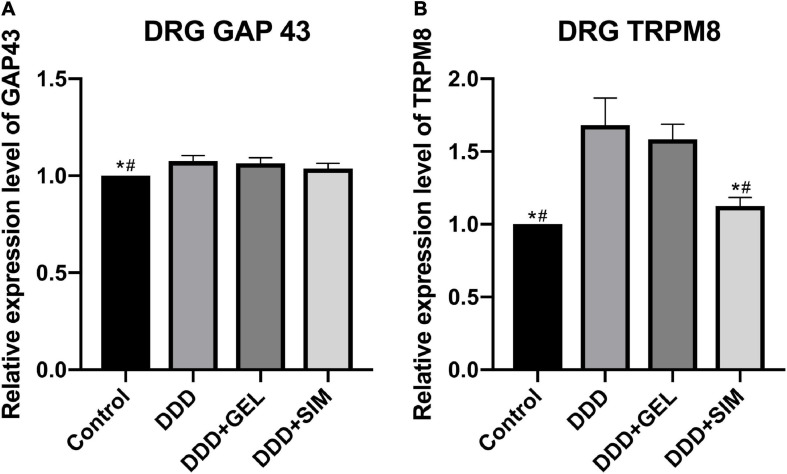
The effect of intradiscal injection of simvastatin on animal DRG nerve mediators. Panel **(A)** is the change of GAP43 in DRG of each group. Panel **(B)** is the change of TRPM8 in DRG of each group; all data are expressed as mean ± standard deviation. *P < 0.05 vs. DDD group, ^#^*P* < 0.05 vs. DDD + GEL group (*n* = 5).

The content of GAP43 in the DRG of the Control group was lower than that of the DDD group and DDD + GEL group (*P* = 0.0029, *P* = 0.0067, Figure 8A). The content of TRPM8 in DRG in the Control group (*P* = 0.0002, *P* = 0.0004) and DDD + SIM group (*P* = 0.0006, *P* = 0.0016) was lower than that of the DDD group and DDD + GEL group (Figure 8B). This shows that intradiscal injection of simvastatin can reduce the cold response.

### The Effect of Intradiscal Injection of Simvastatin on the Skin Nerve Mediators of Rat Hind Paws

**FIGURE 9:**
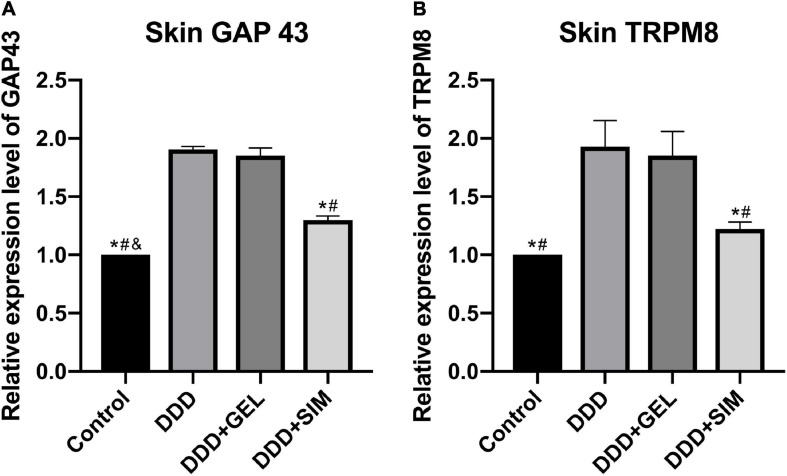
The effect of intradiscal injection of simvastatin on nerve mediators of rat hind paws. Panel **(A)** is the change of GAP43 in the hind paw skin of each group of rats. Panel **(B)** is the change of TRPM8 in the hind paw skin of each group of rats; all data are expressed as mean ± standard deviation, *P < 0.05 vs. DDD group, ^#^*P* < 0.05 vs. DDD + GEL group, ^&^*P* < 0.05 vs. DDD + SIM group (*n* = 5).

The content of GAP43 in the hind paws of rats in the Control group was lower than that in the DDD group, DDD + GEL group, and the DDD + SIM group (*P* < 0.0001, *P* < 0.0001, *P* < 0.0001); GAP43 in the hind paws of rats in the DDD + SIM group The content was lower than that of the DDD group and the DDD + GEL group (*P* < 0.0001, *P* < 0.0001, Figure 9A). The content of TRPM8 in the DRG of the Control group (*P* = 0.0002, *P* = 0.0003) and the DDD + SIM group (*P* = 0.0009, *P* = 0.0017) was lower than that of the DDD group and DDD + GEL group (Figure 9B). These data indicate that intradiscal injection of simvastatin can significantly reduce the growth of nerves in the hind paw of rats and reduce the cold response.

### The Effect of Intradiscal Injection of Simvastatin on the Inflammatory Factors of Rat Intervertebral Disk

**FIGURE 10:**
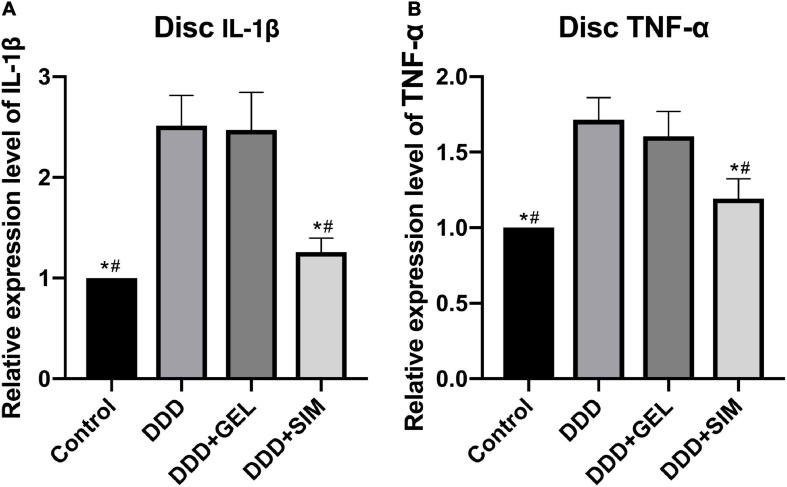
The effect of intradiscal injection of simvastatin on inflammatory factors of rat intervertebral disk. Panel **(A)** is the change of intervertebral disk IL-1β in each group. Panel **(B)** is the change of intervertebral disk TNF-α in each group; all data are expressed as mean ± standard deviation, *P < 0.05 vs. DDD group, ^#^*P* < 0.05 vs. DDD + GEL group (*n* = 5).

The levels of IL-1β in the IVDs of the Control group (*P* = 0.0001, *P* = 0.0002) and DDD + SIM group (*P* = 0.0004, *P* = 0.0005) were lower than those of the DDD group and DDD + GEL group (Figure 10A). The levels of TNF-α in the IVDs of the Control group (*P* = 0.0001, *P* = 0.0003) and DDD + SIM group (*P* = 0.0006, *P* = 0.0023) were lower than those in the DDD group and DDD + GEL group (Figure 10B). These data indicate that intradiscal injection of simvastatin can significantly reduce the inflammation in the IVD of rats.

## Discussion

The development and progression of DDD to DLBP remains unclear and represents an important gap in our knowledge. A key feature of IVD degeneration is the apparent elevation of pro-inflammatory factors and formation of nerves and blood vessels in the disks. These changes have been closely related to the severity of DDD. In this study, we observed highly elevated NF-κ B1, Rela, IL-1 β,TNF-α,GAP43, SP, and CGRP expressions in the disks of DDD groups as compared with the sham groups. This findings is in line with the results observed in IVD tissues extracted from DDD patients (Zhongyi et al., 2015). The new findings of this study include: Compared with the DDD group, the expression of NF-κB1, Rela, IL-1β, TNF-α, GAP43, SP, and CGRP in the IVD of the intradiscal injection of simvastatin group was significantly reduced. More importantly, intradiscal injection of simvastatin can alleviate DLBP and reduce the cold sense reaction of both lower limbs.

Simvastatin were initially used to control and treat patients with hyperlipidemia and hypercholesterolemia (Mundy et al., 1999). Recently, many studies have shown that simvastatin can not only regulate inflammation, but also enhance bone induction, promote bone formation and angiogenesis, and inhibit osteoblast apoptosis and osteoclast generation (Skoglund et al., 2002; Moshiri et al., 2015). Since Mundy et al. (1999) first reported that statins can promote bone metabolism, people have begun to pay attention to the mechanism of statins in human body and verify their anabolism. Zhang and Lin (2008) have shown that simvastatin can promote the cartilage expression in rat IVD cells cultured *in vitro*, and the possible mechanism is that simvastatin stimulates the expression of endogenous BMP-2 in IVD cells. However, the specific mechanism of simvastatin in the treatment of DLBP is still unclear.

Pain behavioral tests are commonly used in animal models as indirect evidence for assessing the severity and progression of pain (Millecamps et al., 2015). It has been reported that cold hypersensitivity occurs after sciatic nerve injury or inflammation of the hind paw (Jasmin et al., 1998; De la Calle et al., 2002). However, there was no change in cold perception in previous models of disk puncture. In the model of this study, cold hypersensitivity can be observed in rats with IVD injury, which is similar to the results of SPARC gene knockout mouse model (Millecamps et al., 2012). This study is the first time to find that intradiscal injection of simvastatin can significantly reduce cold hypersensitivity. The transient receptor potential cation channel responsible for cold perception is a subfamily m member 8 (TRPM8; McKemy, 2007). It can be suggested that simvastatin can reduce the content of TRPM8 to reduce cold hypersensitivity in rats. Our results also showed that there was no significant difference in the threshold of mechanical pain between the left and right hind paws, and there was no significant difference in the weight-bearing capacity of both lower limbs, which indicated that the DLBP model in this experiment would not cause radiation pain in the lower limbs of rats.

The histological staining results showed that the acupuncture disk degeneration occurred 2 weeks after the operation. This is consistent with other studies, which show that disk degeneration begins 2 weeks after injury (Li et al., 2014). The staining results at the 8th week after the treatment of the IVD showed that simvastatin can delay the degeneration of the IVD and repair the shape of the IVD.

It has been reported that the pathological nuclear factor kappa-light chain enhanced activation of B-lymphocyte (NF-κB) is associated with a variety of degenerative diseases, such as osteoarthritis (Berenbaum, 2004), rheumatoid arthritis (Dai et al., 2004), and muscular dystrophy (Acharyya et al., 2007). NF-κB mainly consists of two subunits: RelA/p65 and NF-κB1/p50. NF-κB-mediated inflammation of chondrocytes can lead to progressive extracellular matrix damage (Gu et al., 2016). The activation of NF-κB measured by the upregulated expression of RelA has been reported in IVD tissues extracted from DDD patients (Zhongyi et al., 2015). Blocking nuclear RelA with specific inhibitors can reduce the degree of IVD degeneration in the DDD rat model (Ma et al., 2015), indicating the role of NF-κB in the degeneration of IVD. The results of this study show that simvastatin treatment of DLBP may reduce the activation of the NF-κB pathway, reduce the secretion of IL-1β and TNF-α, and thereby reduce the stimulation of new nerve fibers. This is in contrast to previous studies of NF-κB and its Downstream pro-inflammatory cytokines can cause neuropathic pain consistent (Hartung et al., 2015).

The results of this study suggest that NF-κB may be involved in the regulation of pain-related mediators in the IVD tissue and the growth of nerves in the IVD. In rat models, intrathecal injection of NF-κB inhibitors can reverse neuromechanical pain (Pan et al., 2010). It has been reported that systemic administration of NF-κB inhibitors can reverse inflammatory pain, reduce the expression of SP and CGRP in joints, and the expression of SP and CGRP in dorsal root ganglia (DRG) in animal models of arthritis (Ahmed et al., 2010, 2017). It has been reported that in DDD patients, the sensory nerves innervating degenerative IVD will up-regulate the expression of CGRP and SP (Richardson et al., 2009; Krock et al., 2014). In addition, increased expression of SP was found in human nucleus pulposus (NP) cells stimulated by TNF-α and NP tissues extracted from elderly female DDD patients (Song et al., 2017). Studies have shown that SP and CGRP are the main peptidergic neurotransmitters. SP and CGRP can not only regulate cardiovascular contraction function, but also regulate pain transmission (Mao et al., 1992). Previous studies have shown that CGRP can regulate the activation of NF-κB through the phosphorylation and degradation of IκB in mouse thymocytes (Li et al., 2014). Studies have also confirmed that SP can increase the secretion of cytokines by activating NF-κB (Berenbaum, 2004). These studies confirmed that peptidergic neurotransmitters have feedback regulation effects on NF-κB signaling. According to the results of this experiment, the possible mechanism of intradiscal injection of simvastatin on alleviating DLBP is to reduce the activation of NF-κB, thereby reducing the content of SP and CGRP, and ultimately reducing pain.

In normal lumbar IVDs, the sensory nerve fibers are only distributed on the surface layer of annulus fibrosus (AF). Some scholars first discovered the existence of nerve fibers in the deep layer of the IVD of patients with low back pain (Shinohara, 1970). The essence of IVD degeneration is the imbalance between differentiation, disintegration and synthesis (Sato et al., 1999). As soon as the degeneration begins, the normal stress balance between NP and AF begins to lose, causing the tension of collagen fibers in AF to decrease, which increases the impact load of the endplate cartilage during normal activities, resulting in microfractures and pain (Freemont, 2009). This kind of micro-fracture allows the growth of fibers, blood vessels and nerves to grow into the inner fibrous annulus, nucleus pulposus and even endplate cartilage (Hilton and Ball, 1984). Since then, in the study of patients suffering from IVD pain, some scholars have discovered that the IVD has extensive innervation (Freemont et al., 1997). In our study, GAP43, an index of nerves in the IVD after acupuncture, was significantly increased, but decreased significantly after simvastatin treatment. This shows that intradiscal injection of simvastatin can reduce nerve growth, thereby reducing DLBP. The nerve fibers that grow into the IVD need to pass through the afferent sensory neurons in the DRG to transmit sensory signals to the central nervous system. This experiment found that the content of GAP43 in DRG was significantly increased, which is consistent with previous research conclusions (Miyagi et al., 2014).

There are some shortcomings in this experiment. First of all, female rats were used in this experiment, considering that the proportion of female patients with DLBP is much higher than that of male patients. However, in future experiments, we will increase the comparison of male rats to reduce the bias error caused by gender differences. Secondly, whether simvastatin can delay the degeneration of IVD, but other factors (such as disk size (annulus fibrosus thickness), operator’s proficiency, nutritional status of experimental animals and other unknown factors) can also lead to IVD degeneration. Thirdly, acetone was used to measure the cold pain response in rats. Acetone evaporation is considered not only a measure of thermal hyperalgesia, but also a measure of chemical induced pain sensitivity (van der Wal et al., 2015). This lack of specificity and can not measure the response to specific temperature. Finally, rats were used as subjects in this experiment. However, due to the differences in IVD size, morphology and physicochemical properties of nucleus pulposus cells between human and rats, simvastatin may have different effects on IVD. This study needs to be further verified in human IVDs.

## Conclusion

Simvastatin, on the one hand, inhibits the growth of nerve fibers in the IVD, and on the other hand, significantly reduces the amount of inflammatory factors in the IVD through the NF-KB pathway (inflammatory factors stimulate the new nerves in the IVD) to achieve pain relief. However, the specific mechanism still needs further research. This study provides new strategies and possible targets for the treatment of discogenic low back pain.

## Data Availability Statement

The original contributions presented in the study are included in the article, further inquiries can be directed to the corresponding author/s.

## Ethics Statement

The study was approved by the Animal Care and Use Committee of University of Cincinnati.

## Author Contributions

XH: substantial contributions to the research design, data acquisition, statistical analysis, drafting the manuscript, and revising it critically. CZ, WW, XY, and C-YL: data acquisition and approval of the submitted and final version. ZW: substantial contributions to the research design, data acquisition, and approval of the submitted and final version.

## Conflict of Interest

The authors declare that the research was conducted in the absence of any commercial or financial relationships that could be construed as a potential conflict of interest.
